# A Blind Source Separation Method Based on Bounded Component Analysis Optimized by the Improved Beetle Antennae Search

**DOI:** 10.3390/s23198325

**Published:** 2023-10-08

**Authors:** Mingyang Tang, Yafeng Wu

**Affiliations:** College of Energy and Power, Northwestern Polytechnical University, Xi’an 710129, China; tmy2021100232@mail.nwpu.edu.cn

**Keywords:** blind source separation, bounded component analysis, Beetle Antennae Search

## Abstract

Currently, the widely used blind source separation algorithm is typically associated with issues such as a sluggish rate of convergence and unstable accuracy, and it is mostly suitable for the separation of independent source signals. Nevertheless, source signals are not always independent of one another in practical applications. This paper suggests a blind source separation algorithm based on the bounded component analysis of the enhanced Beetle Antennae Search algorithm (BAS). Firstly, the restrictive assumptions of the bounded component analysis method are more relaxed and do not require the signal sources to be independent of each other, broadening the applicability of this blind source separation algorithm. Second, the objective function of bounded component analysis is optimized using the improved Beetle Antennae Search optimization algorithm. A step decay factor is introduced to ensure that the beetle does not miss the optimal point when approaching the target, improving the optimization accuracy. At the same time, since only one beetle is required, the optimization speed is also improved. Finally, simulation experiments show that the algorithm can effectively separate independent and dependent source signals and can be applied to blind source separation of images. Compared to traditional blind source separation algorithms, it has stronger universality and has faster convergence speed and higher accuracy compared to the original independent component analysis algorithm.

## 1. Introduction

Blind source separation (BSS) is a signal processing method that can obtain the source signals to be processed from the mixed signal observed without confirming the characteristics and quantity of the source signals, as well as the mixing matrix of the transmission channel [1]. Its objective is to reconstruct the source signal as closely as feasible using an estimation method. BSS has important theoretical significance and practical application value in various fields, such as speech, communication, biomedical engineering, and earthquake detection. Although BSS has many characteristics and advantages, it has some limitations. Because of the lack of pre-examined knowledge, the research on blind source separation is based on many assumptions. However, there is a possibility that actual source signals do not meet these assumption conditions, resulting in poor separation effects that affect the spread of blind-sound separation techniques.

Independent component analysis (ICA) algorithms for blind source separation were first developed by A.J. Bell and T.J. Sejnowski in 1995 [2]. They also presented hypothetical conditions for the application of ICA methods. Since then, the research on linear mixed signal separation has been transformed into exploring the objective function of ICA and its optimization method. Until now, the ICA algorithm is still the mainstream algorithm for processing blind source separation. However, in practical environments, some source signals may not meet the assumptions of the ICA method, such as image processing in the field of face recognition, financial impact factors that affect the market, communication-related interference, etc. In recent years, researchers’ interest has been drawn to Bounded Component Analysis (BCA), which offers a new approach to blind source separation. In BCA, the assumptions of ICA are replaced by the compactness and Cartesian separability of the source signals, which are more relaxed than that of ICA and can still be separated without requiring source signals to be independent of each other. After that, the BCA algorithm was improved by other academics. Cruces suggested that the best way to separate a single source signal from a mixed signal is to minimize the convex perimeter function [3]. Erdogan established the concepts of main hyper ellipsoids and boundary hyper rectangles and developed a BCA approach with volume ratio maximization as its main objective [4]. The correlation coefficients between source signals are well tolerated by this approach [5]. The computational complexity of all these BCA approaches is high in terms of the objective function. To solve this problem, Gong et al. [6] proposed a normalized boundary objective function that simplified the objective function of BCA and verified the effectiveness of this method via simulation and experiment. Babatas et al. [7] proposed that the PCA algorithms can be applied to temporal or spatially dependent sources, as well as independent sources. Today, the PCA algorithm has been successfully applied in many engineering fields. Cheng [8] used the PCA algorithm to improve the accuracy of gas turbine noise source recognition, and Tang developed a digital Self-Interference Cancellation algorithm based on bounded component analysis to improve the bit error rate performance of the In-Band Full Duplex system [9].

In conclusion, the research on the blind source separation algorithm based on BCA is fundamentally transformed into an examination of the objective function and its optimization method of BCA. This algorithm is sensitive to initial values and has unstable solution accuracy during the optimization process. For this problem, some scholars propose using swarm intelligence algorithms with stronger global convergence to optimize the objective function. Genetic algorithms were used to solve the BSS problem for the first time by Vetter et al. in 1997 [10]. In 2004, the Particle Swarm Optimization (PSO) technique was effectively applied by Gao et al. [11] to optimize the objective function. In 2009, Zhang [12] proposed a linear BSS method based on the Ant Colony Optimization (ACO) algorithm. In 2016, Li et al. [13] proposed a firefly optimization algorithm based on a new step size adjustment rule and applied it to the BSS problem. In 2018, Chu Dingli et al. [14] optimized the signal’s kurtosis using the whale optimization technique with adaptive weights, which still provided good separation performance at low signal-to-noise ratios. Faramarzi presented a nature-inspired metaheuristic called the Marine Predators Algorithm (MPA) and its application in engineering in 2020 [15]. Li proposed the slime mold algorithm, and Zervoudakis introduced a new method called the Mayfly Algorithm (MA) to solve optimization problems in the same year [16,17]. A BSS approach based on the genetic lion swarm optimization algorithm was proposed by Zhang et al. in 2021 [18], which effectively applied the algorithm to the blind separation of image signals while increasing the accuracy of the algorithm.

This article proposes an improved BCA algorithm based on BAS-BCA to solve the problems of slow convergence speed and unstable precision in the BCA algorithm. Without any prior knowledge, this algorithm can extract and separate independent and dependent source signals from mixed signals. The BAS algorithm avoids entering local optima during the iteration process by providing a step decay factor. The objective function of the BCA algorithm is optimized using the improved BAS algorithm, enhancing the efficiency and precision of the method. Simulation experiments prove that this algorithm not only has stronger universality but also has faster convergence speed and more stable precision than traditional blind source separation algorithms and BCA algorithms.

To summarize, the major contributions of this paper are given as follows:

(1) An improved BCA algorithm based on the Beetle Antennae Search (BAS) algorithm is proposed to address the shortcomings of conventional blind source separation algorithms.
(2) A step decay factor is introduced to the BAS algorithm, which avoids entering local optima during the iteration process.
(3) Simulation results show that the BAS-BCA algorithm successfully separates the dependent and independent source signals. This algorithm not only has stronger universality but also has faster convergence speed and more stable precision than traditional blind source separation algorithms and BCA algorithms.

The rest of the paper is organized as follows: Section 2 introduces the BSS model and the basic BCA algorithm; Section 3 introduces the improved BAS algorithm and the proposed BAS-BCA algorithm; the performance of this method was demonstrated via simulation analysis in Section 4; The conclusion is shown in Section 5.

## 2. Theories of BSS Based on BCA

### 2.1. BSS

Assuming that the transmission of the signal is instantaneous, the mixed signals can be expressed as follows [19,20]:

(1) $$x_{i}(t)=\sum_{j=1}^{n}\;h_{ij}s_{j}(t)+n_{i}(t)$$

where $x_{i}(t)$ denotes the mixed signals received by sensors,$s_{j}(t)$ denotes the source signals, $n_{i}\left(t\right)$ denotes the observation noise of the *i*-th sensor, and the above equation can be expressed as Equation (2) using a matrix.

(2) $$x\left(t\right)=As\left(t\right)+n\left(t\right)$$

where $s(t)={\left[s_{1}(t),s_{2}(t),s_{3}(t)\ldots s_{m}(t)\right]}^{T}$ denotes an m-dimensional column vector,$\;x(t)={\left[x_{1}(t),x_{2}(t),x_{3}(t)\ldots x_{n}(t)\right]}^{T}$ and $n\left(t\right)$ denote n-dimensional column vectors, $A=\left(a_{1},\;a_{2},\;a_{3}\ldots a_{n}\right)$ denotes the mixing matrix, and *a*_1_ = *h*_1*j*_, *a*_2_ = *h*_2*j*_, *a*_3_ = *h*_3*j*_, …, *a_n_* = *h*_1*j*_, *j* = 1, 2, 3, …, *m*, assuming we believe that the noise was processed to a negligible level before blind source separation.

(3) $$x\left(t\right)=As\left(t\right)$$

From the above equation, it can be seen that the process of blind source separation is actually conducted by estimating the mixing matrix *A* when x(t) is known and the mixing matrix *A* and the source signal s(t) are unknown, and by deriving a matrix *M*, such that *M* carries out a linear transformation of x(t) to satisfy Equation (4).

**Figure 1:**
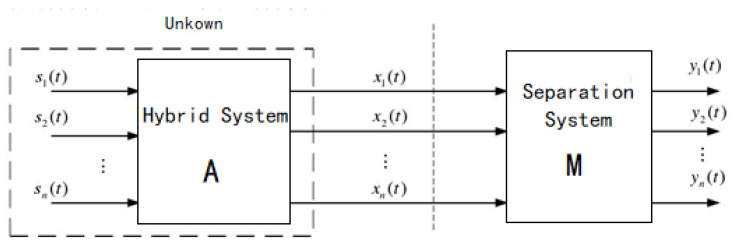
Model of linear mixed signal separation.

(4) y(t)=Mx(t)

where y(t) is the estimate of the source signal s(t), which is the source signal separated from x(t) and *M* is called the separation matrix. Thus

(5) $$y\left(t\right)=Mx\left(t\right)=M\times As\left(t\right)=Ws\left(t\right)$$

where *W* is positive definite and is called the complex separation matrix. In particular, when *m = n*, *M* is the inverse matrix of *A*. The whole model of linear mixed signal separation is shown in Figure 1.

### 2.2. BSS Based on BCA

Compared to the ICA algorithm, the biggest advantage of the BCA algorithm is that it can separate non-independent signals, which brings more universality to the blind source separation algorithm. Its basic principle is similar to the ICA algorithm, and it also requires some necessary assumptions [21,22]:

(1) Assume that the mixing matrix A has rank n, i.e., column full rank, i.e., the number of sensors should be greater than or equal to the number of source signals.
(2) Assume that the distribution of source signals is bounded.
(3) Assume that the branch set S, consisting of the joint distribution of all source signals, can be expressed by the Cartesian product of the branch set Si of each source signal:
  (6) $$S=S_{1}⊗S_{2}⊗\cdots ⊗S_{n}$$
  where *n* is the number of source signals and ⊗ denotes the Cartesian product.

Assumptions (2) and (3) are weaker and more loosely constrained compared to the assumptions of ICA. Assumption (3) is automatically satisfied when the signals are independent of each other, so the blind source separation algorithm based on BCA has stronger generalizability.

With the help of the analysis above, it is clear that the BCA algorithm’s steps are to find the separation matrix *M* when the mixed signal *X* is known, and then multiply *M* by *X* to determine *Y*, which is the estimated value of the source signal *S*. Then, the process of the BCA algorithm can be simply summarized as solving the separation matrix to approximate the independent source signal, and the specific approximation method is actually an optimization search of the established objective function. Thus, the algorithm can be simply described as follows:

BCA algorithm = optimization criterion + optimization-seeking algorithm

In fact, this criterion is to construct some objective function and then use this function for separation. The selection of the objective function determines the statistical performance of the algorithm, and the optimization algorithm determines the convergence performance, storage requirements, and stability of the algorithm. In this paper, the normalized boundary objective function is selected as follows:

(7) J(y)=R^(y)E{y2}

y denotes the separation signal, $E\left\{y^{2}\right\}$ denotes the variance of y, and R^(y) is the boundary operator.

(8) R^(y)=maxy−min(y)

where $\max\left(y\right)\;and\;\min\left(y\right)$ denote the maximum values of y and the minimum values of y. 

The objective function J(y) is minimized if and only if the global vector *W* is a perfect separator, that is, when *W* has only one non-zero component.

The proof is given below.

Combining with the previous Equation (5) and assumption (3), Equation (8) can be written as

(9) R^(y)=W+−W−(maxs−min(s))

where s denotes the source signals, $\max\left(s\right);\min\left(s\right)$ denote the maximum values of s and the minimum values of s; $W_{+}$ denotes extracting positive components of *W* while setting others to zero; and $W_{-}$ denotes extracting the negative components of *W* while setting others to zero. We define $\forall =diag\left(\max\left(s\right)-min(s)\right)$, and then combining the matrix theory knowledge, we can obtain [6] the following:

(10) R^(y)=W∀1=ℓ1

We define ℓ=W∀. The vector ℓ can be written as

(11) $$\ell =\sum_{i=1}^{N}\ell_{i}\varepsilon_{i}$$

where $\varepsilon_{i}$ denotes the basic unit vector. Define ${\varepsilon_{i}}^{\prime }=sign(\ell_{i})\varepsilon_{i}$ and ${\ell_{i}}^{\prime }=\left|\ell_{i}\right|/\sum_{i=1}^{N}\ell_{i}$, then we can obtain

(12) $$\ell =\sum_{i=1}^{N}\left|\ell_{i}\right|{\varepsilon_{i}}^{\prime }=\sum_{i=1}^{N}\left|\ell_{i}\right|\sum_{i=1}^{N}\;{\ell_{i}}^{\prime }{\varepsilon_{i}}^{\prime }=\|\ell {\|}_{1}\sum_{i=1}^{N}\;{\ell_{i}}^{\prime }{\varepsilon_{i}}^{\prime }$$

Define $\hat{\;s}=\forall^{-1}s$, thus $s={\forall \hat{s}}^{\;}$, we can obtain

(13) $$E\left\{y^{2}\right\}=\frac{W\forall \hat{s}{\hat{s}}^{T}\forall^{T}W^{T}}{N}=\frac{\ell \hat{s}{\hat{s}}^{T}\ell^{T}}{N}$$

where *N* is the number of source signals, Equation (7) can be written as

(14) $$J\left(y\right)=\frac{1}{\|\ell {\|}_{1}\sum_{i=1}^{N}\;{\ell_{i}}^{\prime }{\varepsilon_{i}}^{\prime }\sqrt{\hat{s}{\hat{s}}^{T}/N}}$$

So, the objective function J(y) is minimized if and only if $\ell_{i}$ has only one non-zero component, i.e., the global vector g has only one non-zero component.

Combining with the previous Equation (4), and assuming that *Q* is the covariance matrix of the observed signal *x*, Equation (7) can be written as follows:

(15) J(M)=R^(Mx)MQMT

## 3. Blind Source Separation Algorithm Based on BAS-BCA

### 3.1. BAS

**Figure 2:**
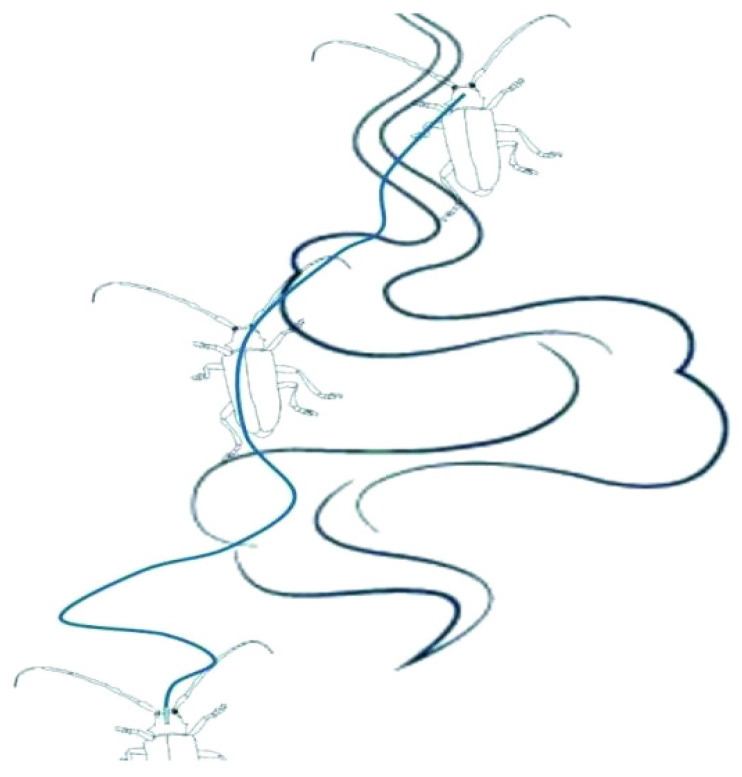
Overview of the BAS algorithm.

The BAS algorithm is a heuristic algorithm mainly based on the foraging behavior of the beetle [23], which can be effectively used in optimization problems [24,25,26,27]. The beetle uses its left and right whiskers to detect the flavor concentration of food when foraging. If the left whisker finds a food with a higher taste concentration, it goes to the left side, and vice versa. As seen in Figure 2, during the entire movement process, its position is continuously updated and altered until it ultimately reaches the location of the food.

The overall algorithm flow is as follows:

1. An initial position defined as *X*_0_ is given before beetle foraging.
2. Determine the location of the beetle antennas. To ensure the randomness of the aspen search direction, a random factor is defined as *P* with the following expression.
  (16) $$P=\frac{rand(n,1)}{\left\|rand(n,1)\right\|}$$
  where rand(n,1) denotes an n-dimensional random number between 0 and 1.

The position of the left antenna of the beetle is defined as *X_L_*, the position of the right antenna of the beetle is defined as *X_R_*, and they are related to the position *X*_0_ of the beetle as follows:

(17) $$X_{L}=X_{0}+D\times P$$

(18) $$X_{R}=X_{0}-D\times P$$

where *D* is the distance between the left antenna of the beetle and the right antenna of the beetle.

3. The left antenna *X_L_* and the right antenna *X_R_* are brought into the adaptation function equation to obtain the magnitude of the detected food taste concentration, which is used to update the position of the beetle.
  (19) $$X_{k+1}=\left\{\begin{array}{c} X_{k}+E\times \rho \times \frac{X_{L}-X_{R}}{\left\|X_{L}-X_{R}\right\|}\;f\left(X_{L}\right)<f(X_{R})\\ X_{k}-E\times \rho \times \frac{X_{L}-X_{R}}{\left\|X_{L}-X_{R}\right\|}\;f(X_{L})\geq f(X_{R}) \end{array}\right.$$
  where $X_{k}$ denotes the position of the beetle in the k-th cycle, $f\left(\cdot \right)$ denotes the fitness function, and ρ denotes the travel step length of the beetle. In this paper, we improve on the basic BAS algorithm by introducing a step decay factor *E* and using adaptive weights [28] as the decay factor.
  (20) $$E=\cos\left(\frac{\pi \times k}{2\times M}+\frac{\pi }{2}\right)+1$$
  where *k* denotes the number of cycles and *M* is the maximum number of iterations.
4. Enter the loop process, and when the set maximum number of iterations *M* is reached, or the adaptation value reaches the set requirement, stop the iteration and output the optimal result.

The pseudo-code of BAS is provided in Algorithm 1.
**Algorithm 1** BASInputs: maximum number of iterations *M*, fitness function $f\left(\cdot \right)$, step decay factor *E*, line progress length ρ of the beetle, distance *D* between the left antenna and the right antenna of the beetle
Initialize the location of beetle *X*While *k* < *M*Calculate the random factor using Equation (16)Calculate the position of the left and right antenna of the beetle using Equations (10) and (18)If $f\left(X_{L}\right)<f(X_{R})$Update the position of the beetle using Equation (19)Update the step decay factor using Equation (13)ElseUpdate the position of the beetle using Equation (19)Update the step decay factor using Equation (20)end ifend whileoutput optimal results


In order to test the performance of this algorithm, a simulation of the six standard test functions given in Table 1 was performed, including the single peak test function (F1 to F3) for testing the ability and convergence accuracy of the algorithm to extract group information, and the multi-peak testing function (F4 to F6) for testing the ability of the algorithm to explore information other than the population and solve complex optimization problems. In Table 1, DIM denotes the function dimension, *scope* represents the value range of x, and *f_min_* indicates the ideal value of each function.

BAS is compared against two algorithms in the experiment, namely the Grey Wolf Optimization Algorithm (GWO) [29] and the Whale Optimization Algorithm (WOA) [30]. For all ten algorithms, the population size N = 30 and the total number of iterations T = 500.

Table 2 shows the optimal fitness value (BEST), the standard deviation (STD), and the running time (TIME), tested by three algorithms, such as BAS, under six test functions in Table 1, in which the time unit is seconds. Each algorithm was performed separately 30 times to minimize the error, and all experiments were conducted on a laptop equipped with an Intel (R) Core (TM) i7-6500 CPU at 2.50 GHz and 8 GB of RAM.

As shown in Table 2, it can be seen that this BAS algorithm has basically converged to the minimum value in the calculation of functions F1, F2, F4, F5, and F6, and the standard deviation is also close to 0, proving the high stability of the algorithm. Although the F3 function did not converge to the minimum value, overall, the BAS algorithm has better convergence performance than other algorithms. As for the calculation time in Table 2, the BAS algorithm of this paper has a medium execution time. According to the data in the table, the test time for BAS under the three test functions of F2, F3, and F4 is less than that of the GWO and WOA.

### 3.2. BAS-BCA

This paper proposes a blind source separation algorithm based on BAS-BCA, which has the following advantages compared with the current mainstream blind source separation algorithm: First, the constraint of the a priori assumptions of this algorithm is more relaxed, and there is no need to ensure that the source signals are independent of each other, thus enhancing the universality and accuracy of this algorithm, which can cope with more complex environments and more diverse source signals; second, it uses the population intelligence algorithm. Secondly, the BAS algorithm is used for the optimization of the objective function, and the number of beetles in the BAS algorithm is only one, so its computation will be greatly reduced, which makes the optimization faster and more robust; finally, the step decay factor is introduced so that the beetle does not miss the best value point when approaching the objective, which improves the optimization accuracy.

The main flow of the algorithm is as follows:

1. Pre-processing of the received mixed signal, including de-averaging and pre-whitening.
2. Parameter initialization. The observed mixed signals (in the case of three signals) are used as the position information of the individual beetle X = [X1, X2, X3], the maximum number of iterations M, the step decay factor E, the travel length of the aspen ρ, the distance between the left antenna and the right antenna of the beetle D.
3. Determine the left antenna X_L_ and right antenna X_R_ of the beetle according to Equations (16) and (17).
4. Calculate the objective function J(W) as the fitness of the beetle by Equation (15).
5. Update the position of the aspen $X_{k+1}$, according to Equation (19).
6. Update the step decay factor using Equation (20).
7. If the algorithm satisfies the termination condition, go to step 8; otherwise, repeat step 3 until it is satisfied.
8. Output separation matrix W and estimate the source signal.

**Figure 3:**
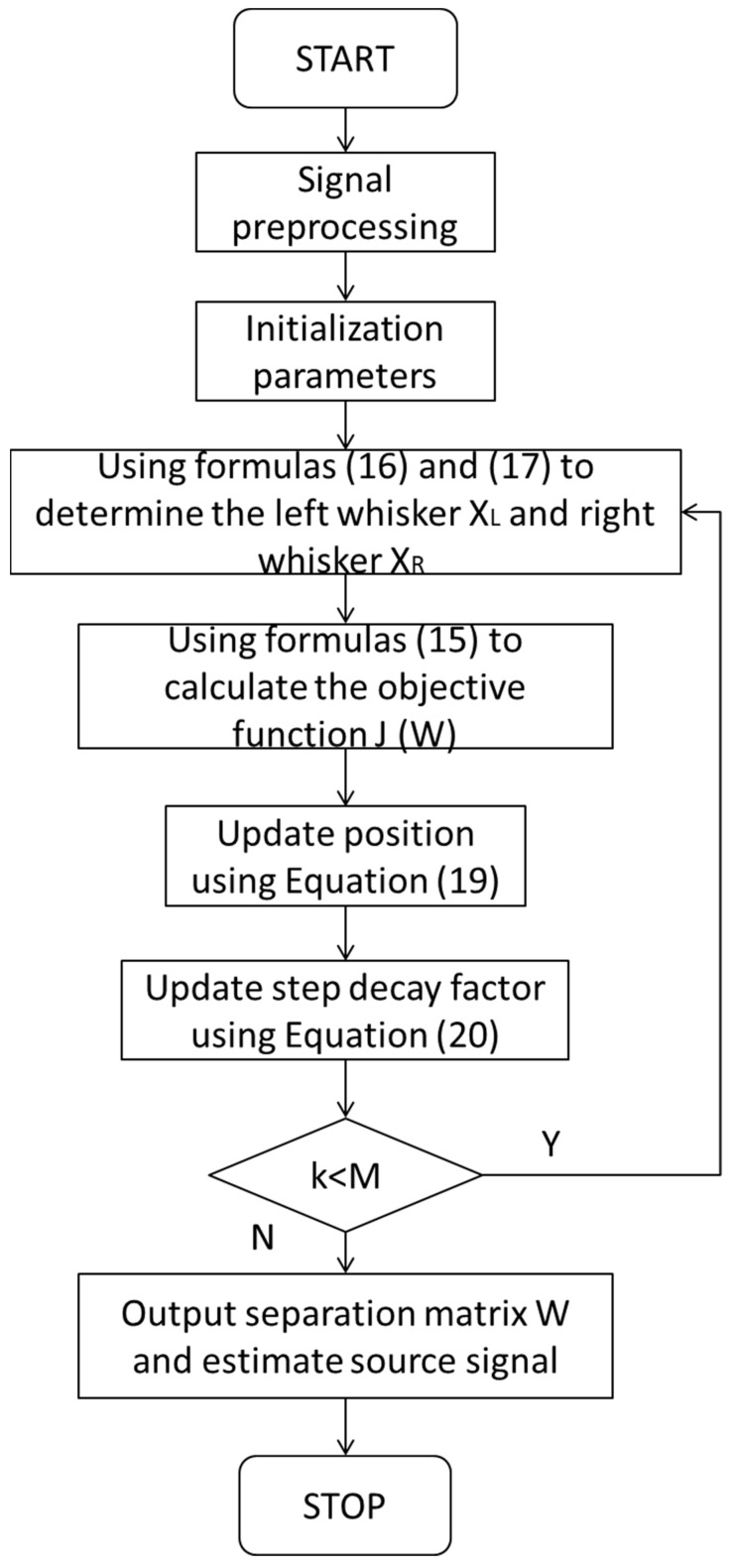
The flow of BAS-BCA algorithm.

Figure 3 shows the flow chart of the BAS-BCA algorithm.

## 4. Simulation and Experimentation

### 4.1. Separation of Independent Source Signals

**Figure 4:**
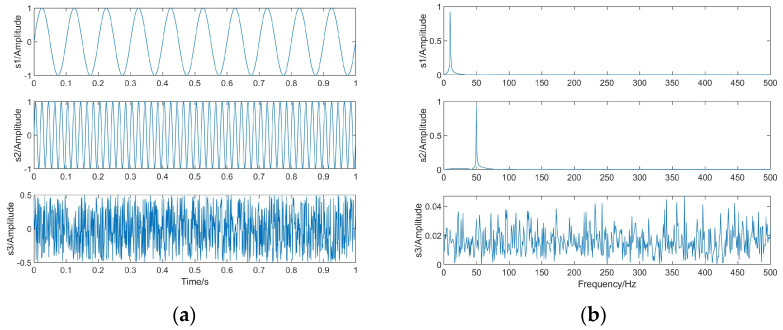
The source signals. (**a**) Waveforms. (**b**) Spectra.

This simulation experiment is intended to demonstrate the usability of the proposed algorithm for independent source signals. Three independent source signals are generated in Python: s1, s2, and s3. s1 is a sinusoidal signal *s*1 = *sin*(20 *πt*) with a frequency of 10 Hz; s2 is a sinusoidal signal *s*2 = *sin*(100 *πt*) with a frequency of 50 Hz; s3 is a random signal *s*3 = *rand*(1,*L*) − 0.5; L is the length of the signal L = 1000. The number of sample points Ne is also 1000. Their waveforms and spectra are shown in Figure 4.

**Figure 5:**
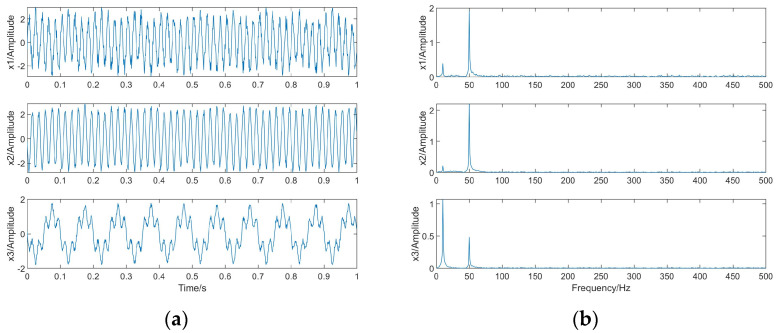
The observed signals. (**a**) Waveforms. (**b**) Spectra.

The linear mixing matrix A = {0.3763 2.0243 −1.3216; −0.2270 −2.3595 −0.6361; −1.1489 −0.5100 0.3179} is a third-order square matrix, and the source signal is linearly mixed to obtain the observed signals x1, x2, x3, whose waveforms and spectrograms are shown in Figure 5.

**Figure 6:**
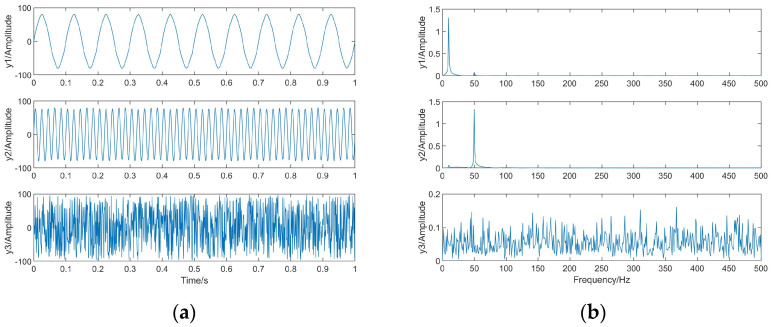
The separated signals. (**a**) Waveforms. (**b**) Spectra.

The waveforms and spectra of the separated signals are shown in Figure 6 after separating the observed signals with the BAS-BCA algorithm.

And obtain the separation matrix *W* = {−0.7862 0.6179 −0.0033; −0.6177 −0.7858 0.02762; −0.0144 −0.0237 −0.9996}. In order to see the separation effect more objectively and accurately, the correlation coefficient ρ between the separated signal and the source signal is chosen as the evaluation criterion in this paper [31], as shown in Equation (21), and the results are shown in Table 3 as follows:

(21) $$\rho_{\;}=\frac{\mathrm{cov}\left(s,y\right)}{\sqrt{\mathrm{cov}\left(s,s\right)\mathrm{cov}\left(y,y\right)}}$$

where cov(·) denotes the variance, *s* denotes the source signal, and *y* denotes the separated signal. From the knowledge of probability theory, it is known that the similarity coefficient has the following properties:

1. $\left|\rho \right|\leq 1$, represents the similarity of s and *y*, and the closer to 1, the more similar,
2. $\left|\rho \right|=1$, which means that *s* and *y* are completely similar,
3. $\left|\rho \right|=0$, which means *s* and *y* are not similar at all.

**Table 3:** Correlation coefficient.

	y1	y2	y3
**S1**	0.97990255	0.01395991	0.00630897
**S2**	0.00404252	0.96984297	0.00778653
**S3**	0.00741474	0.00730825	0.94597251

From Table 3, it can be seen that the correlation coefficients of s1 and y1, s2 and y2, and s3 and y3 are all close to 1, which corresponds to Figure 4 and Figure 6. From Figure 4 and Figure 6, it can be seen that three independent source signals are successfully separated based on the BAS-BCA algorithm.

**Figure 7:**
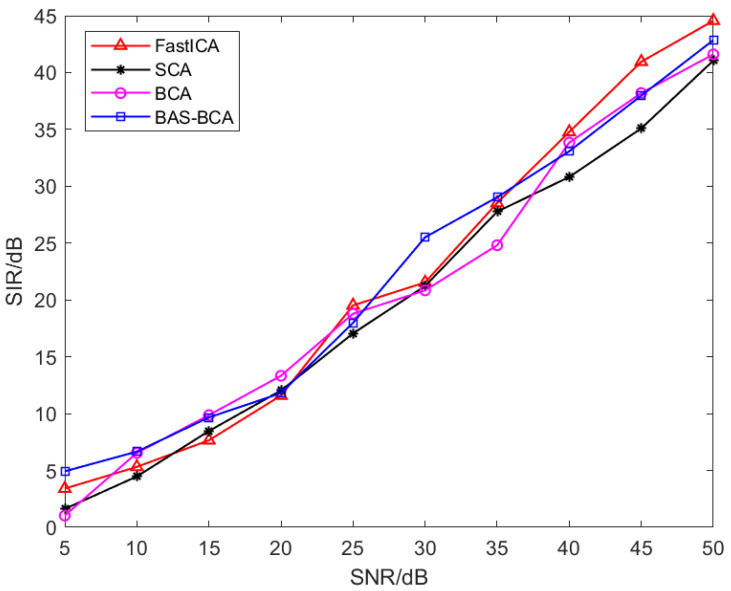
Comparison of separation accuracy for independent signals.

As a comparison, the signal interference ratio (SIR) [32] is also selected to evaluate the separation results by using FastICA, SCA, and the BCA algorithm from the literature [31]. The equation of SIR is shown in Equation (22) as follows:

(22) $$SIR=-10lg\frac{{\left\|y_{i}-s_{i}\right\|}^{2}}{{\left\|s_{i}\right\|}^{2}}$$

where $y_{i}$ is the i-th signal in the separated signal *y* and $s_{i}$ is the i-th signal in the source signal *s*. Signal processing-related knowledge has shown that the separation is improved by increasing the SIR value. We made 10 sets of experiments with different e signal-to-noise ratios (SNRs), and the results are shown in Figure 7.

As can be seen from Figure 7, all algorithms’ separation accuracy increases with increasing SNR. The average NSIR of the BAS-BCA algorithm, the BCA algorithm in [4], SCA, and the FastICA algorithm are 21.95, 20.89, 19.96, and 21.78, respectively. The BAS-BCA algorithm has the best separation accuracy, followed by the FastICA algorithm. Therefore, the superiority of the proposed algorithm in independent signal separation is proved.

### 4.2. Separation of Dependent Source Signals

This section demonstrates via simulation experiments and comparisons that the algorithm in this paper is also suitable for blind source separation of dependent source signals, while the separation effect is better and faster than the traditional blind source separation algorithm.

Three source signals that are correlated with each other are generated by Python. They obey the copurn-t distribution, and their correlation matrices are as follows:

$$R=\left[\begin{array}{ccc} 1 & q & q^{2}\\ q & 1 & q\\ q^{2} & q & 1 \end{array}\right]$$

where the value of q is a random number between 0 and 1.

**Figure 8:**
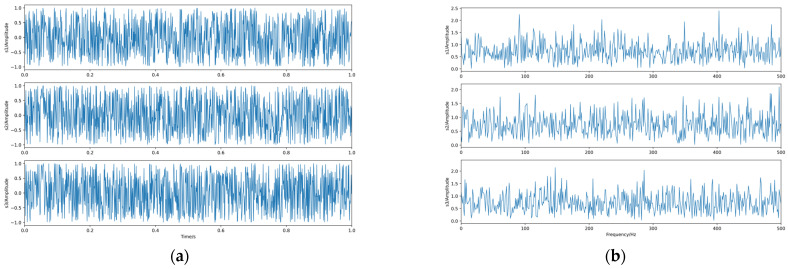
The source signals. (**a**) Waveforms. (**b**) Spectra.

Firstly, *q* is taken as 0.625 for the experiment, and again, 1000 sample points are taken, and the generated waveform and spectrum of the dependent source signal are shown in Figure 8.

**Figure 9:**
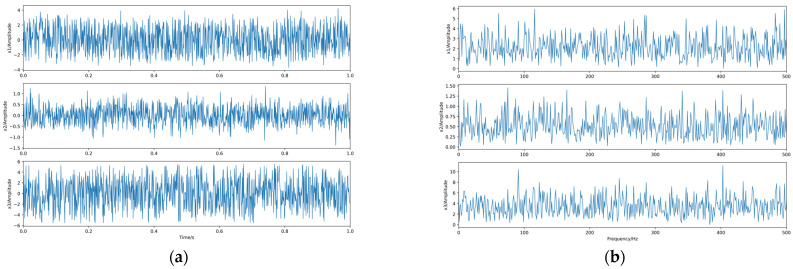
The observed signals. (**a**) Waveforms. (**b**) Spectra.

The linear mixing matrix is still A. The source signal is linearly mixed to obtain the observed signals x1, x2, and x3, whose waveforms and spectrograms are shown in Figure 9.

**Figure 10:**
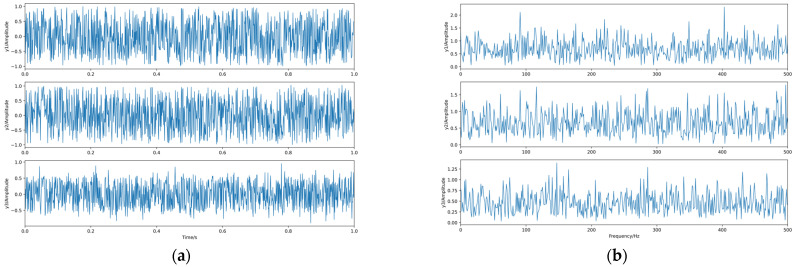
The separated signals. (**a**) Waveforms. (**b**) Spectra.

The waveforms and spectra of the separated signals are shown in Figure 10 after separating the observed signals with the BAS-BCA algorithm.

And obtain the separation matrix *W* = {−1.39184926 −0.11501416 0.02704677; 0.2524785 −0.67398223 −0.12270163; −0.04869351 0.01061255 −0.68229682}. The correlation coefficients between the separation signal *y* and the source signal *s* are shown in Table 4.

From Table 4, it can be seen that the correlation coefficients of s1 and y1, s2 and y2, and s3 and y3 are all close to 1, which corresponds to Figure 8 and Figure 10. It can be seen that the BAS-BCA algorithm successfully separates the three dependent source signals.

Next, the two traditional blind source separation algorithms, FastICA and SCA, as well as the BCA algorithm from the literature [4], are used to perform blind source separation of the mixed signals. In order to objectively and quantitatively compare the performance of the three algorithms, the performance index (PI) and the running time were selected for the study, in addition to the similarity coefficients and SIR.

The PI evaluation index mainly uses the difference between the mixing–separation matrix and the generalized alignment matrix to measure the blind source separation effect, as shown in Equation (23).

(23) $$PI=\frac{1}{N(N-1)}\sum_{i=1}^{N}\left\{(\sum_{i=1}^{N}\;\frac{\left|g_{ij}\right|}{\underset{i}{max}|g_{ij}|}-1)+(\sum_{j=1}^{N}\;\frac{\left|g_{ij}\right|}{\underset{j}{max}|g_{ji}|}-1)\right\}$$

where *N* represents the number of source signals, $\underset{i}{max}|g_{ij}|$ represents the maximum value of |g| in line *i* of the matrix *G*, $g_{ij}$ is the (*i*, *j*)th element of the matrix *G*, and *G* is the product of the separation matrix *W* and the mixing matrix *A*, which is the global matrix. Smaller PI values indicate better separation.

To more obviously demonstrate the advantages of the algorithm in this paper in terms of runtime, 500,000 sample points were taken for the algorithm comparison experiment, and other conditions were kept constant. The results are shown in Table 5. It can be seen that the signal separated by the BAS-BCA algorithm has the highest correlation coefficient with the source signal, and the SIR value is also the largest and produces the smallest PI value, so the separation effect of the BAS-BCA algorithm is the best, and the running time of the algorithm is also shorter than the BCA algorithm.

**Figure 11:**
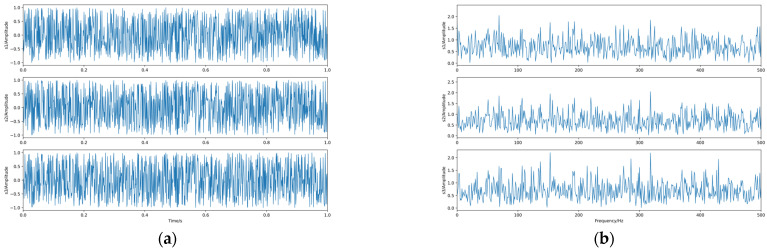
The source signals. (**a**) Waveforms. (**b**) Spectra.

Then, we conducted an experiment of highly dependent sources separation. *q* is taken as 0.912, and again, 1000 sample points are taken, and the generated waveform and spectrum of the dependent source signal are shown in Figure 11.

**Figure 12:**
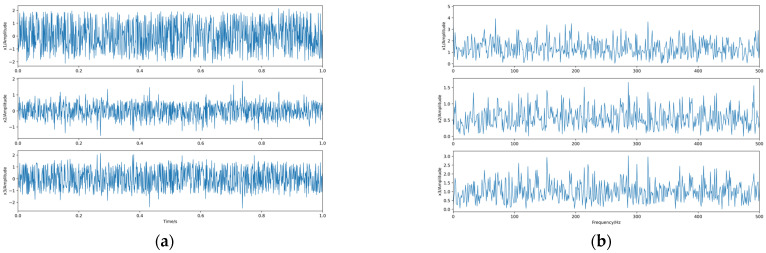
The observed signals. (**a**) Waveforms. (**b**) Spectra.

The linear mixing matrix is still A. The source signal is linearly mixed to obtain the observed signals x1, x2, x3, whose waveforms and spectrograms are shown in Figure 12.

**Figure 13:**
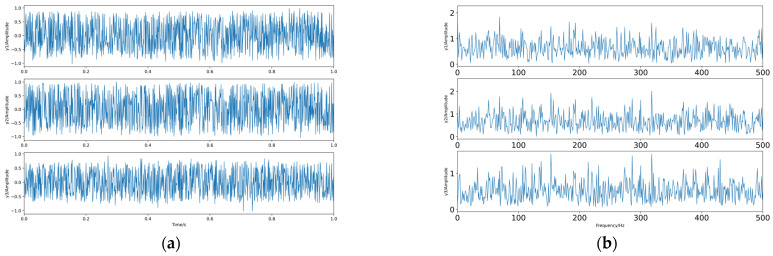
The separated signals. (**a**) Waveforms. (**b**) Spectra.

The waveforms and spectra of the separated signals are shown in Figure 13 after separating the observed signals with the BAS-BCA algorithm.

And obtain the separation matrix *W* = {−0.57989843 0.27412716 0.30326697; 0.25319743 −1.08609897 −0.28978132; −0.15634155 −0.50392014 −0.71771854}. The correlation coefficients between the separation signal *y* and the source signal *s* are shown in Table 6.

From Table 6, it can be seen that the correlation coefficients of s1 and y1, s2 and y2, and s3 and y3 are all close to 1, which corresponds to Figure 11 and Figure 13. It can be seen that the BAS-BCA algorithm successfully separates the three dependent source signals. Comparison results with other algorithms are shown in Table 7.

It can be seen that the BAS-BCA algorithm still performs the best compared to other algorithms. However, compared to the PI and SIR values of the experiment with q set to 0.625, the current experiment has a higher PI value and lower SIR value. It can be seen that the performance of the BAS-BCA algorithm in highly dependent source separation has decreased, but compared to other algorithms, it has a better separation effect.

**Figure 14:**
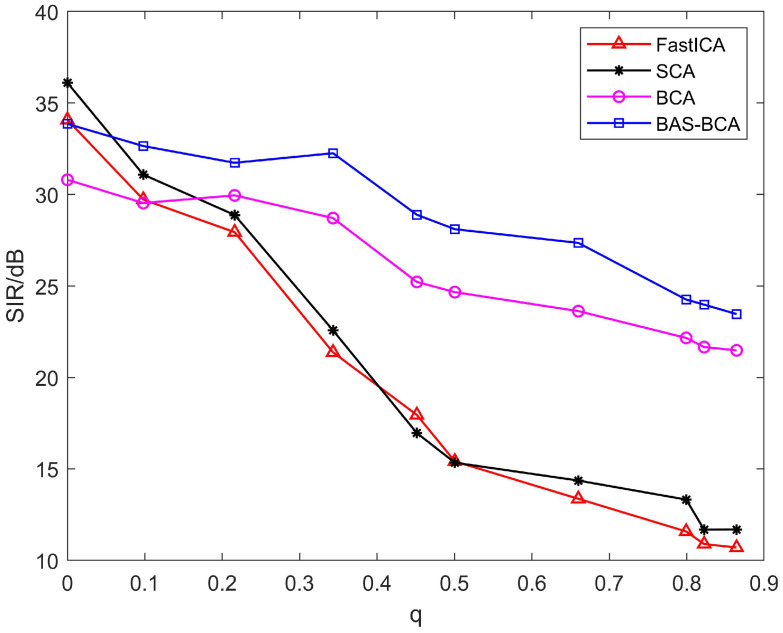
Comparison of correlation signal separation accuracy.

In order to further explore the effect of correlation between source signals on the separation effect of blind source separation algorithms, the SIR values corresponding to each algorithm are obtained by selecting 10 different *q*-values, as shown in Figure 14.

As can be seen from Figure 14, the SIR values of all algorithms decrease as q increases, i.e., the higher the correlation between source signals, the lower the accuracy of the separation algorithms. The average SIRs of the BAS-BCA, BCA, SCA, and FastICA algorithms are 28.65, 25.78, 20.19, and 19.3, respectively. The separation results of the BAS-BCA algorithm are significantly better than those of the BCA algorithm, and the average SIR of the BAS-BCA method increased by 2.87. Traditional blind source separation algorithms such as FastICA and SCA are only applicable to mutually independent source signals, so the separation results are poor. Such algorithms have the best separation accuracy when the sources are independent, i.e., q is close to zero. When the source signals are not mutually independent, the separation accuracy of the FastICA and SCA algorithms decreases rapidly with the increase in the relevant parameter q. The average SIR of the BAS-BCA algorithm is 9.35 and 8.46 higher than that of the FastICA and SCA algorithms, respectively. Therefore, the superiority of the BAS-BCA algorithm in the separation of dependent source signals is verified.

### 4.3. Blind Source Separation of Images

Three grayscale images are selected as the source signals S, each with 512 × 512 pixels, and a 3 × 3 mixing matrix A is randomly generated to mix the source signals to obtain the observed signals X. The BAS-BCA, BCA, SCA, and FastICA algorithms are used to separate the observed signals, respectively. The correlations among the source signals are shown in Table 8.

**Figure 15:**
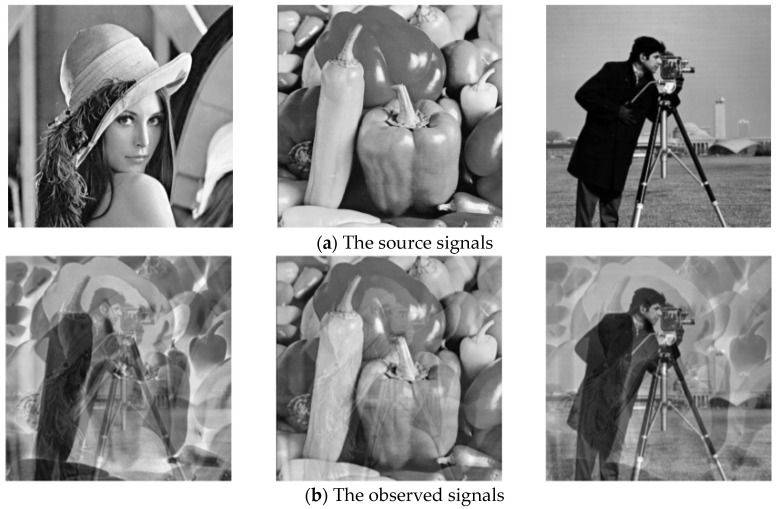

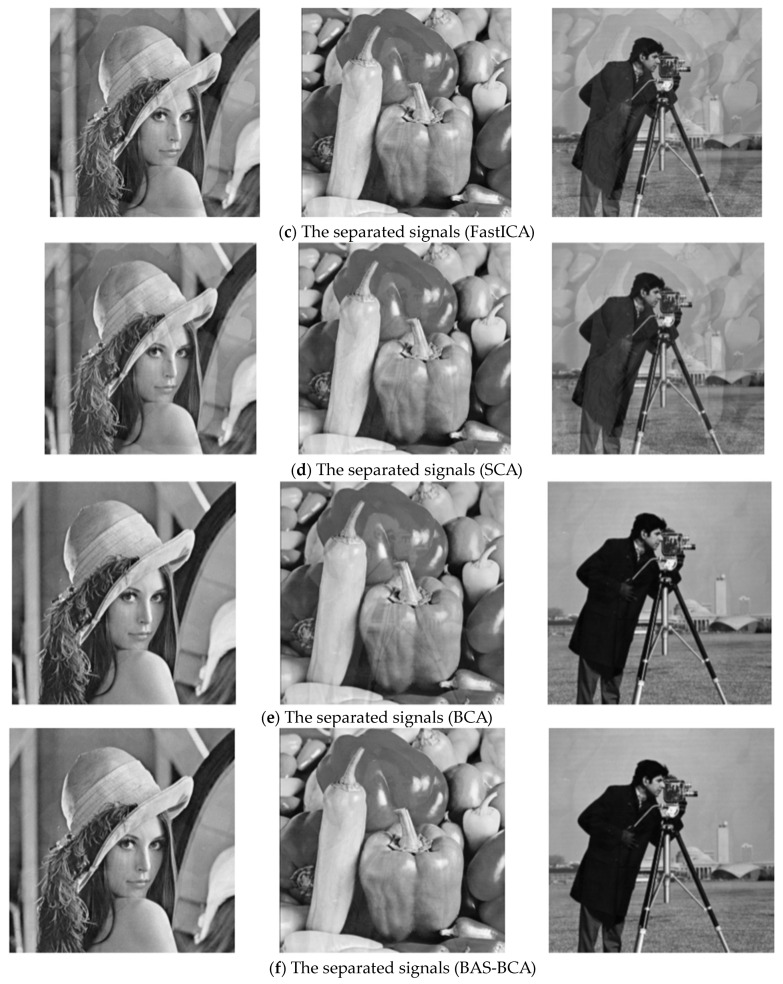
Effect drawing of image signal separation. (**a**) The image of source signals; (**b**) the image of observed signals; (**c**) the image of FastICA separated signals; (**d**) the image of SCA separated signals; (**e**) the image of BCA separated signals; (**f**) the image of BAS-BCA separated signals.

Figure 15 shows the simulation results, and Table 9 compares the correlation coefficient, PI, run time, and SIR of the separated signals.

According to Figure 15, the three images separated by the BAS-BCA algorithm are the closest to the original images, whereas the images separated by the other methods have different degrees of ambiguity. A more objective look at Table 9 shows that the BAS-BCA algorithm’s average similarity coefficient is the one that is closest to 1, while also having the smallest PI value and highest SIR value. This indicates that the BAS-BCA algorithm proposed in this paper has higher accuracy and better performance in processing blind source separation of images, as well as a shorter running time.

## 5. Conclusions

In this paper, we propose a blind source separation algorithm based on BAS-BCA, which is optimized by the BAS algorithm and then combined with the BCA algorithm for blind source separation, thus enhancing the universality, as well as the accuracy of the blind source separation algorithm while having better operation efficiency and separation accuracy. The algorithm introduces a step decay factor in BAS to avoid falling into the local optimum and improve the convergence performance and then applies the improved BAS algorithm to the optimization process of the BCA algorithm to make the whole algorithm have higher operation efficiency and better optimization accuracy. The constraints of the prior assumptions of this algorithm are more relaxed, and there is no need to ensure that the source signals are independent of each other, so it can cope with more complex environments and more diverse source signals.

In order to verify the effectiveness and superiority of the present algorithm, simulation, and analysis experiments are conducted. First, the separation simulation of independent sources was performed, and the similarity coefficients of their separated signals and source signals were 0.97990255, 0.96984297, and 0.94597251, which were all close to 1, proving that the present algorithm is suitable for blind source separation of independent sources as other blind source separation algorithms. Then, the simulation of blind source separation was carried out for dependent signal sources, and the separation effects were compared with those of FastICA and SCA, two traditional blind source separation algorithms, and the BCA algorithm. This algorithm is also applicable to the separation of dependent source signals and has better separation accuracy and running efficiency than the other three algorithms, as evidenced by the similarity coefficient, performance index (PI), running time, and signal interference ratio (SIR) of the separated signals. We concluded that the lower the independence of the source signal, the worse the effect of the traditional blind source separation algorithm. Finally, this algorithm was applied to the blind source separation of images and compared with the other three algorithms, which shows that the performance of this algorithm in image separation also has a better separation effect.

## Figures and Tables

**Table 1:** Test function.

Number	Function	DIM	*scope*	*f_min_*
**F1**	$f_{1}(x)=\sum_{i=1}^{D}\;x_{i}^{2}$	30	[−100, 100]	0
**F2**	$f_{2}(x)=\sum_{i=1}^{D}\left|x_{i}\right|+\prod_{i=1}^{D}\left|x_{i}\right|$	30	[−10, 10]	0
**F3**	$f_{3}(x)=\sum_{i=1}^{D-1}\left[100{\left(x_{i}^{2}-x_{i+1}\right)}^{2}+{\left(x_{i}-1\right)}^{2}\right]$	30	[−30, 30]	0
**F4**	$f_{4}(x)=\frac{1}{4000}\sum_{i=1}^{D}\;x_{i}^{2}-\prod_{i=1}^{D}\;cos(\frac{x_{i}}{\sqrt{i}})+1$	30	[−600, 600]	0
**F5**	$F_{5}(x)=\sum_{i=1}^{n}\left[x_{i}^{2}-10cos(2\pi x_{i}+10)\right]$	30	[−5.12, 5.12]	0
**F6**	$f_{12}(x)=20+e-20exp(-0.2\sqrt{\frac{1}{D}\sum_{i=1}^{D}\;x_{i}^{2}})-exp(\frac{1}{D}\sum_{i=1}^{D}\;cos(2\pi x_{i}))$	30	[−32, 32]	0

**Table 2:** Comparative analysis of performance of 3 intelligence algorithms.

Number	BAS			GWO			WOA		
	BEST	STD	TIME	BEST	STD	TIME	BEST	STD	TIME
**F1**	8.43 × 10^−9^	1.32 × 10^−9^	0.124	1.28 × 10^−19^	3.39 × 10^−19^	0.176	0.279	0.13	0.085
**F2**	4.47 × 10^−14^	0.348	0.092	2.01 × 10^−9^	3.41 × 10^−9^	0.182	1.24 × 10^−16^	4.82	0.098
**F3**	22.63	7.06 × 10^−7^	0.096	0.269 × 10^2^	0.601	0.213	4.14 × 10^2^	5.36 × 10^2^	0.103
**F4**	1.01 × 10^−5^	1.11 × 10^−4^	0.085	1.84 × 10^−3^	4.12 × 10^−3^	0.219	0.038	0.016	0.089
**F5**	−1.92 × 10^−3^	0.136	0.081	0.398	0.545 × 10^2^	0.198	1.64 × 10^−2^	0.462 × 10^2^	0.079
**F6**	2.35 × 10^−3^	0.542	0.076	2.19 × 10^−4^	3.54 × 10^−4^	0.192	5.09 × 10^−3^	1.61	0.073

**Table 4:** Correlation coefficient.

	y1	y2	y3
**S1**	0.95674313	0.39900721	0.04960461
**S2**	0.47858537	0.98699327	0.47763681
**S3**	0.35747411	0.50449069	0.97789969

**Table 5:** Data of separation performance evaluation index.

	FastICA	SCA	BCA	BAS-BCA
**similarity coefficient**	0.61478832 0.79113256 0.86792361	0.75382446 0.83249712 0.84562887	0.94112682 0.89978596 0.96120685	0.99913226 0.97777913 0.98947889
**PI**	0.37858537	0.33259634	0.18382564	0.09763681
**SIR**	17.78995818	18.58967436	23.98567492	27.52189291
**Time**	19.36	20.21	29.68	25.11

**Table 6:** Correlation coefficient.

	y1	y2	y3
**S1**	0.99705728	0.86008656	0.66929815
**S2**	0.87889169	0.99727447	0.82707136
**S3**	0.75027519	0.89507997	0.98287344

**Table 7:** Data of separation performance evaluation index.

	FastICA	SCA	BCA	BAS-BCA
**similarity coefficient**	0.52367416 0.71235987 0.79534238	0.76545382 0.63541287 0.77852536	0.93564258 0.90312354 0.95238421	0.99705728 0.99727447 0.98287344
**PI**	0.49896372	0.39986341	0.21368547	0.15236846
**SIR**	11.6689403	12.9658332	22.6854931	24.6235874
**Time**	20.68	21.93	31.42	26.57

**Table 8:** Correlation coefficient.

	s1	s2	s3
**s1**	1	0.27858537	0.34960461
**s2**	0.27858537	1	0.12389069
**s3**	0.34960461	0.12389069	1

**Table 9:** Data of separation performance evaluation index.

	FastICA	SCA	BCA	BAS-BCA
**similarity coefficient**	0.81583695 0.80563229 0.83652718	0.85426875 0.82156371 0.84685932	0.96189537 0.92678932 0.95345874	0.98256749 0.96585374 0.99136457
**PI**	0.29356417	0.27998659	0.14567789	0.11268894
**SIR**	18.1235845	20.2314567	23.2589671	28.9358791
**Time**	21.56	24.13	33.15	28.35

## Data Availability

The data presented in this study are available upon request from the corresponding author.
